# On-pitch concussion management: a view of healthcare professionals in elite football

**DOI:** 10.1136/bmjsem-2025-002805

**Published:** 2025-11-30

**Authors:** Ryan Baker, Jac Palmer, Gareth Irwin, Sean Connelly, Genevieve Williams

**Affiliations:** 1 Public Health and Sport Sciences, University of Exeter, Exeter, UK; 2 Cardiff School of Sport, Cardiff Metropolitan University, Cardiff, UK; 3 Football Association of Wales, Cardiff, UK

**Keywords:** Concussion, Football, Qualitative Research, Sports medicine, Soccer

## Abstract

Collecting an account of practitioners’ lived experiences of on-pitch concussion management in football provides real-world insight into areas for improvement, and future research direction. Previous research suggests that healthcare professionals (HCPs) in football are confident with on-pitch concussion recognition; however, some players are still returned to the field to later be removed with a concussion.

**Objectives:**

The aim of this study is to understand the on-pitch assessment procedure of HCPs and factors that may influence whether or not a player is removed following a suspected head injury in football based on semistructured interviews.

**Methods:**

10 elite-level pitch-side HCPs participated in a semistructured interview. Recorded interview transcripts were analysed via reflexive thematic analysis. This process generated four key themes: time, formalised procedure for identifying concussion, interpretation of behaviour and stakeholder actions.

**Results:**

This study provides evidence that HCPs in football feel that they do not have enough time to conduct a thorough concussion assessment. Assessments are currently subjective and rely on the sideline healthcare staff’s relationship with the player to determine if they are acting normally. The HCPs value seeing the mechanism of injury and advocate the use of video replays. Temporary concussion substitutes would allow more time for assessment and for the use of assessment tools such as the Sport Concussion Assessment Tool.

**Conclusions:**

Based on the current findings, research should focus on developing multimodal testing batteries to detect concussion on-pitch, while football should consider implementing video analysis at all levels and temporary concussion substitutes.

**WHAT IS ALREADY KNOWN ON THIS TOPIC**

- Healthcare professionals in football are confident with recognising a concussion pitch-side and are given enough time by referees to conduct an assessment on-pitch. In the 2018 FIFA World Cup, 50% of the players eventually removed with a head injury were returned to play prior to removal.

**WHAT THIS STUDY ADDS**

- Pitch-side healthcare professionals would like the introduction of temporary concussion substitutes. Healthcare professionals value seeing the mechanism of injury and knowing the player to determine if a player should be permanently removed.

**HOW THIS STUDY MIGHT AFFECT RESEARCH, PRACTICE OR POLICY**

- This research could help to inform rule changes in football regarding temporary concussion substitutes and highlights the necessity for multimodal assessment batteries to detect changes in function due to concussion.

## Introduction

 The 2022 consensus statement on concussion in sport states that to avoid further possible injury, any player should be removed from the field of play if there is suspicion of a concussion. Signs such as loss of consciousness, poor balance, confusion and behavioural changes all warrant immediate removal from the field.^1^ However, even on the international football stage, there are instances where a player is not removed from the field of play following the on-pitch assessment but is later removed. For example, in the 2018 FIFA World Cup, three of the six players removed due to a head injury were initially returned to play following the on-pitch evaluation.^2^ Evidently, there are still challenges in removing a player from the football pitch in the incidence of a concussive injury.

Following a head impact event, an on-pitch healthcare professional (HCP) can request an optional 3 min match interruption to assess the affected player on-pitch.^3^ The FIFA Medical Network Diploma states that the focus of the on-pitch assessment of a player who has suffered head trauma is to assess the player for concussion or a more severe head or cervical spine injury. The Sport Concussion Assessment Tool sixth edition (SCAT6)^1 4^ is currently the assessment tool recommended by FIFA and in its entirety takes 10–15 min to complete. The on-pitch assessment examination is based on the ‘Immediate Assessment/Neuro screen’ portion of the SCAT6. Following this 3 min assessment, the player is either substituted and removed from play, where the complete SCAT6 assessment can be undertaken, or the player is cleared to continue playing in the same game. HCPs in the UK can access the Advanced Trauma and Medical Management in Football course,^5^ which covers head injury management and the FA concussion guidelines.

Governing bodies in football are aware that there is a concern regarding concussions and are frequently updating rules and regulations to increase player safety. One of the most notable interventions to date was the introduction of an additional permanent concussion substitute during the 2020/2021 season of the English Premier League. However, this intervention resulted in no significant difference in the frequency or duration of medical assessments for concussion injury.^6^ Still, based on a medical opinion, Gouttebarge *et al*^7^ recommended that the time window for concussion assessment in football should be extended to 10 mins and should be conducted in a distraction-free environment, aligning with the consensus statement on concussion in sport guidelines.^1^

Rosenbloom *et al*^8^ examined the options of professional football medical staff (HCPs) using questionnaires and found that 88% believe that players ‘sometimes’ (48%) or more frequently (‘very often’—39% or ‘always’—7%) under-report symptoms to avoid removal. A minority of the medical staff reported they sometimes (27%) or often (13%) felt coaches or managers attempted to influence their decision making in respect to the removal of a player with concussion.^8^ Furthermore, the authors reported that medical team staff overall felt they were given enough time to assess for a concussion on-pitch; however, only 16% of the medical staff responded that they were ‘always’ given enough time. While medical team staff reported being confident with concussion recognition,^8^ we still do not know why some injured players are returned to the field following an evaluation for concussion.

The aim of this study is to understand the on-pitch assessment procedure of HCPs and factors that may influence whether or not a player is removed following a suspected head injury in Football based on semistructured interviews. The findings of this study will provide evidence from HCPs in football as to the challenges faced when evaluating a player following a head contact event under the current rules in football with the purpose to inform future rule changes.

## Methods

### Study design

This study employed a qualitative descriptive approach,^9 10^ framed within a critical realist framework. This design was selected as a means of capturing the accounts of participants’ lived experiences while acknowledging the existence of a reality that can be imperfectly understood through interpretation. Semistructured interviews were employed to capture participants’ personal accounts of on-pitch concussion management. Data were analysed using reflexive thematic analysis,^11^ consistent with the interpretive flexibility of qualitative descriptive work within a critical realist framework.

### Eligibility criteria and sampling

Inclusion criteria included HCPs working in the English Premier League, England Football League SkyBet Championship, or Men’s or Women’s UK national teams. The participants were required to be involved in the immediate recognition and/or management of head injuries on-pitch. As recommended by Rodríguez-Teruel and Daloz,^12^ this study took advantage of purposive sampling as the participants were required to be working for clubs in elite-level football, with participants contacted via email. Data saturation was considered to be reached when no new responses to questions occurred in a series of two consecutive interviews.^13^

### Equality, diversity and inclusion

Based on our purposive sampling approach, the sample reflects the population working for clubs in elite-level football. The author team is made up of mixed genders, people from Lesbian, Gay, Bisexual, Transgender, Queer or Questioning, Intersex, Asexual or Agender, and 2Spirit (LGBTQIA2S+) communities, junior and senior researchers with different disciplinary backgrounds and job roles. Inclusivity was considered in data collection by allowing flexible dates and times for participants to complete the interview.

### Data collection

Semistructured interviews were conducted using a standardised script, where prompting was given if a participant answered with a closed response. Each participant took part in one interview which was conducted by the lead researcher. Participants were informed that all data gathered would be confidential and pseudo-anonymised for the purposes of the study.

The interview began with the participant being presented with a hypothetical scenario in which there has been an incident where a player falls to the ground, holding their head. Further information regarding the scenario was available should the participant ask for it; for example, there was no visible head wound or tonic posturing.

Interview questions were designed by the research team to explore four research topics:

1. What is the information available to medical staff before assessing a player with a potential head injury?
2. What aids the decision-making process of medical staff when determining whether or not a player needs to be removed due to a head injury?
3. What challenges do medical staff encounter when assessing whether a player should be removed due to a head injury?
4. If a player remains on the field, what is the postdecision protocol?

### Data analysis

Interviews were conducted and recorded via Microsoft Teams (Microsoft Teams 2021, Microsoft) and transcribed verbatim and coded by the lead author via Microsoft Word (Microsoft Word 2021, Microsoft). All transcripts were checked against recordings for accuracy, and a participant’s own transcript was available for them to review.

Transcripts were analysed in a six-phase process of reflexive thematic analysis as outlined by Braun *et al*.^11^ The process started with ‘familiarisation’ with the dataset, through repeated reading and rudimentary note-taking. This was followed by an initial process of ‘code generation’, identifying or advancing ‘tagging’ data of interest to a code. This process was repeated to ensure a robust and systematic set of codes. Coding was followed by candidate theme generation, which grouped codes together. Coding and theme generation were performed iteratively and collaboratively, thus facilitating the reflexivity of the analysis. These codes were then ‘refined’ and ‘defined’ inductively against the data set. The final phase of the process is known as the ‘write-up’, which draws on data extracts and associated interpretations to form a comprehensive analytic narrative. To enhance reflexivity and analytic depth, the second author was used as a critical friend during this process. Their role was to offer alternative perspectives and challenge assumptions as interpretations of the data evolved.

This study was situated within a critical realist paradigm, reflecting the context-dependent and applied nature of sports medicine. Critical realism deems that an objective reality exists and that one’s perception of this reality is influenced by social, contextual and professional interpretations.^14^ This approach is aligned with the aims of the study to explore how HCPs perceive and act within real-world, complex environments where multiple factors influence on-pitch concussion management. This process was completed in accordance with Braun *et al*^11^ and satisfies the ‘big tent’ criteria for excellent qualitative research set out by Tracy^15^ due to the relevant and timely nature of the research, researcher reflexivity, practical implications and procedural ethics upheld throughout the study.

### Researcher background

The lead researcher was a male research assistant, specialising in concussion, funded by Football Association Wales. The overarching project aim was to develop a quantitative pitch-side concussion assessment tool to aid the decision-making of pitch-side HCPs when determining whether or not a player needed to be removed from play after suffering a head impact event. They had no previous healthcare or medical training or experience.

### Patient and public involvement

Patients and the public were not formally involved in defining the research questions, methods and dissemination of the current results. This research was informed by stakeholders in football including National Governing Bodies and HPCs, in order to ascertain the process by which HCPs understand the on-pitch assessment procedures and factors that may inform clinical decision-making for removal from play following a suspected head injury for the purpose of informing future rule changes in football.

## Results

10 participants were interviewed before data saturation occurred. A total of six team doctors, one consultant physician, one medical director, one physiotherapist and one head of physiotherapy were interviewed. Participants had an average of 10.1±9.1 years (ranging from 1 to 32 years) of experience in football medicine. Six participants had previous experience as a general HCP, three were previously employed from rugby union and one had only worked in football medicine. Interviews lasted 35.8±9.5 min.

Based on the thematic analysis, four key themes developed: ‘time’, ‘formalised procedure for identifying concussion’, ‘interpretation of behaviour’ and ‘stakeholder actions’. These four themes and the subsequent subthemes with quotes exemplifying these subthemes are shown in table 1.

**Table 1:** Themes and subthemes that developed from interviews, and quotes to exemplify subthemes

Time	
On-pitch assessment time	*I'm not always 100% satisfied that in the time frame that I've been given that we've been able to make the right decision*. *I think the way that it could be made better for us is probably giving us more time, I think 3 to 5 min sometimes is not enough* *They want a quick answer. Can this player continue? And I'm being radioed through. Can you give us an answer? Can you give us an answer? And actually, like I say, space clarity and just that little bit of extra time is probably what’s needed*. *Under the current stipulations, with the amount of time that you're allowed and the amount of leeway that you're allowed to make the decisions, I don't think that’s a feasible achievement to get their boots off to do this*.
Comparison to procedures in collision sports	*Like the process they have in rugby, whereas the medical team gets to take the player off for a 10-minute period or however long it is and behind the scenes away from the crowds away from the ref they get that time extended time with the player. You can do a more comprehensive assessment, and you don't have pressure of anyone else talking to you at that time*. *Whether we adopt a similar rule to rugby where you get roll on, roll off subs for that period because you know if you need a 10 minute assessment, your team are down a man and you know you do feel a bit of pressure as a medic for pulling people off for that, especially if you concede a goal, you don't want to be the one on the sideline*. *If those players had a head injury assessment like you have in rugby 10 minutes in the changing room, away from it or sit down, show them the video footage, let the adrenaline drop and their symptoms would have evolved there*.

Formalised procedure for identifying concussion	
Mechanism of injury	*Looking at them as they go down, if they're not putting their arms up to protect themselves. It’s almost ah well, I’m not going to even bother testing orientation, you know they’re just coming off*. *My main thing then is looking at whether they protect themselves or not. And whether they put their arms out when they do go to ground*. *How quickly is the referee waving you on? Are there other players waving you on?*
On-pitch tests	*We would automatically put them into mills. And so, the first priority is to make sure they're alive and there’s no problem. You then be checking for any spinal injury and which is a standard protocol*. *Once you're in mills, you have to follow a protocol essentially where the patient has to respond to you appropriately and they have to do certain things that you ask of them. So, number one, it slows everything down and it buys me a bit of time and number two it gives me more information because I can see whether that player is able to respond appropriately. I get more of a feel for how agitated they are* *The five Maddocks questions, which are pretty subjective, and I think a lot of patients can fly through that*. *I do still use modified Maddocks test not to rule out or exclude concussion, but I think it’s a nice way to get an idea about their orientation, but appreciate that that you know it’s just an added screening tool*

Interpretation of behaviour	
Tacit knowledge	*Once you’ve been doing it for a while, you just know, you got a gut feeling that something isn't right because it comes with experience* *Anything out of the ordinary. A shake of the head and that widening of the eyes almost trying to orientate themselves*.
Relationships	*But it’s the way that they say it and that they try to close it off the conversation. “I’m absolutely fine. I wasn’t knocked out”, not realizing that’s massively important.So they will downplay any questions* *So just trying to get an idea and if they're oriented time person, place and whether they're responding normally, whether they got normal behaviour for them*. *I think you know if you can spot if they're not ‘being themselves’ fairly quickly. I have covered sports and there've been injuries like this before where I haven't known that personality where you get drafted in at the last moment and it does make life trickier.Yeah, you get a sixth sense after you've done it for so long that this particular person is not right*.

Stakeholder actions	
Symptom presentation	*You know there’s a lot of, like lying on the floor trying to potentially demonstrate to the referee that they have been fouled*. *Underreporting is probably more likely or under recognition rather than being deliberately manipulative*. *There are some that definitely underplay their symptoms, just because they want to stay on the pitch*.
Perceived pressure on decision-making	*if it’s a big game, FA Cup final, whatever you know they're going to want to carry on pressure from coaches. I guess if you've got a big crowd, from the crowd as well, you know, if this very important player on the sideline*. *Sometimes I've had refs coming up and saying, right, you know, you've been with them one min, you barely getting through the Maddocks question check. “Are they okay? Can you take them off?” And I've not finished my assessment*.

### Time

#### Subtheme 1: on-pitch assessment time

When asked how interviewees would improve the current on-pitch assessment, 9 out of 10 participants indicated that they would like more time to conduct their assessment. Participants remarked that 3–5 min is not long enough to make an accurate diagnosis of whether a player is safe to remain on the pitch when concussion is suspected. One participant stated, “I’m not always 100% satisfied that in the time frame that I’ve been given that we've been able to make the right decision”.

Reasons for players passing the initial concussion assessment but later needing removal from play were frequently that the player later reported symptoms, when the effect of epinephrine had worn of, or the participant believed that the symptoms had been exercise-induced. HCPs sense that concussion symptoms evolve over time, which some noted is a fairly unique characteristic of the injury (eg, the effect of other musculoskeletal injuries, such as a pulled muscle, is immediately evident).

#### Subtheme 2: comparison to procedures in collision sports

HCPs believed that the diagnosis process could be improved if there were rolling concussion substitutes, or they were able to take the player away from the situation on pitch without necessarily substituting or removing them permanently. Comparisons were made between HCPs in rugby being allowed on the pitch immediately while play goes on around them, whereas in football there must be a stoppage in play and the HCPs invited onto the field by the fourth official.

### Formalised procedures for identifying concussion

#### Subtheme 1: mechanisms of injury

All HCPs identified that the main information they had would be line of sight observation of the mechanism of injury. The reaction of the fellow players and the referee was also mentioned as signs of severity for players’ injury.

Every participant was concerned with the mechanism of injury, with a number saying it was very important to their overall decision-making process. It was deemed helpful to view the mechanism of injury and to view how the player protected themselves when they fell. It was stated that “not having visual information is a significant limitation”. Without a direct line of sight to the incidence, participants are reliant on their colleagues, other players, the referee, or the player themselves to recount the incident, one participant stated, “*you’re stuck*” and they are “*reliant on the 3-minute assessment*”. The use of video replay was deemed very useful by those who had access; however, the provision is not available at all stadiums or levels of the game.

#### Subtheme 2: on pitch tests

The on-pitch assessment was common across most participants and generally included an initial cervical spine assessment, a test of orientation (either conversational or Maddocks questions), a test of co-ordination (finger-to-nose), and an assessment of players vision, hearing, balance and speech. These tests were typically chosen as they form part of the SCAT-6 protocol, which provided confidence that practitioners could defend their actions if necessary.

### Interpretation of behaviour

#### Sub theme 1: tacit knowledge

Key on pitch assessments were identified as oculomotor function, balance and co-ordination, as they are difficult to mask. One participant claimed *“the eyes will tell you everything*” indicating non-verbal communication, such as eye contact, or a “glazed over” look, as identifiers they use to distinguish injured players.

Some participants indicated they would like more objective and quantifiable tests. However, one participant remarked that computer-based tests are useless on game-day, due to the length of time required to complete these types of assessment.

#### Subtheme 2: relationships

Knowing the player was identified as an important factor in the identification of injury. Participants reported that their assessment included subjective measures, such as, “are they [acting like their] normal [selves]?” and are they “responding to questions [as they would] normally?”. Some participants stated that a secondary function of the tests was to assess if a player could follow instructions. Some participants indicated through experience they developed a ‘sixth sense’ or ‘gut feeling’ that they know when something is not right with a player. HCPs expressed that having a relationship with the player allowed them to assess whether they were acting or responding normally based on non-verbal communication. One participant stated, “*So I know the girls quite well, so it’s quite easy for me to spot when one of them’s a bit off. So and so is usually really chatty, she’s quiet, it’s not normal or someone’s really emotional, really irritable, and that really helps as the team doctor. But sometimes you don’t have that luxury if you’re just covering a match, you don't know any of them. So, they might tell you they feel alright. Actually, everyone else would be like she’s not right*”. In the instance that HCPs did not know the player, they stated being more likely to be cautious and remove the player.

HCPs indicated that players who return to play following a suspected head injury are monitored for roughly 10–15 min after the incident. During this time, HCPs reported looking for any signs that the players’ performance has dropped, for example, are they unable to hold the offside line, or whether there is any noticeable change in their personality.

### Stakeholder actions

#### Subtheme 1: symptom presentation

Relying on players to disclose their symptoms was identified as problematic. A number of participants claimed they felt players were likely to under-report their symptoms, with three of those believing participants may also over-report symptoms. Players may under-report as they want to stay on the field or they may not recognise that they are concussed. Players may also not know the symptoms of concussion to report them or not know the severity of concussion. One participant stated, “*underreporting is probably more likely or under recognition rather than being deliberately manipulative”*. Difficulties with reporting injuries were also identified as “when you’ve had a knock to the head, you’re going to have a headache”, similarly players may rub their head or have blurred vision due to contact around their eyes.

On the other hand, it is believed players may over-act to attempt to ‘buy a foul’ or ‘delay the game’. Indeed ‘diving’ or ‘feigning injury’ was highlighted, where it was stated that feigning injury increased the difficulty of quickly determining if the player is truly injured. One participant described this feigning as a “typical football injury, where there might not have been any trauma, but they’ve gone down holding their head or their face”.

#### Subtheme 2: perceived pressure on decision-making

Five participants discussed feeling pressure from either the players themselves or from the coaches/managerial staff to allow a player to remain on the field.

Four participants alluded to facing pressure from the referee to quickly decide if the player needs removing so the game can continue. A good relationship with match officials was seen as a key factor to allow the HCPs to enter the game quickly and be provided time to assess a head injury.

## Discussion

The aim of this study is to understand the on-pitch assessment procedure of professional HCPs and factors that may influence whether or not a player is removed following a suspected head injury in football. Through semistructured interviews, this study was conducted across football leagues in the United Kingdom to gain insights from 10 HCPs working in the English Premier League, England Football League SkyBet Championship, or Men’s or Women’s UK national teams. Based on thematic analysis, four themes were identified: ‘time’, ‘formalised procedures for identifying concussion’, ‘interpretation of behaviour’ and ‘stakeholder actions’. Overall, the HCPs interviewed expressed that they would like to have more time to decide whether or not to remove a potentially concussed player. This time is needed as players may attempt to hide their symptoms from HCPs to remain on the field; therefore, they use a combination of formalised assessments and observations of normal behaviour to determine if a player needs to be removed.

Based on the semistructured interview approach, this study suggests HCPs feel they do not have enough time on-pitch to determine if a player needs removing from the field of play. Every participant interviewed indicated that they would like more time to conduct a concussion assessment. Some specifically indicated that they would like to see the introduction of temporary concussion substitutes in Football. Often reference was made to a desire to see concussion substitution rules similar to those in Rugby Union.^16 17^ This finding is in contrast to Rosenbloom *et al*^8^ who reported that overall HCPs in football felt that officials gave them enough on-pitch time to assess for concussion.^8^ However, only 16% of the medical staff in the Rosenbloom *et al*^8^ study reported ‘always’ being given enough time to complete the concussion assessment, indicating that time can be an issue for HPCs. For example, based on the answers to the question ‘Do referees and officials give you the time needed to assess for concussion pitch-side’, ‘very often’ and ‘sometimes’ were interpreted as ‘reporting having enough time’,^8^ presumably based on the Likert scale criteria. Whereas in our semistructured interview approach, the medical professionals responded they would like more time to ensure that players don’t return to play and develop concussion-like symptoms. Similarly, the previously stated question by Rosenbloom *et al*^8^ could be interpreted as ’do the referees give you the time available within the rules of the game’. Conducting an interview could be considered more accurate than a questionnaire^8^ as there is less likelihood of misinterpretation of questions. In summary, our findings clearly show that HPCs would want more time to conduct a thorough assessment.

HPCs interviewed in this study sense that concussion symptoms are likely to evolve over time—which some noted is a fairly unique characteristic of the injury (eg, the effect of other musculoskeletal injuries, such as a pulled muscle, is immediately evident). Participants are identifying a phenomenon that is known clinically^18^ and that has been reported by injured athletes themselves. For example, Olson *et al*^19^ found that 17% of athletes in their study reported that concussive symptom onset occurred >15 min following head impact.^19^ Since concussion symptoms may take time to appear, HCPs in football staff face challenges in deciding if to permanently remove a player after a potential head injury within the 3 min on-pitch window, highlighting the potential need for more time or the option to temporarily substitute the player until symptoms become evident.

A common difficulty faced by doctors is a player who may under-report their symptoms to remain on the field. Rosenbloom *et al*^8^ found that 93% of professional HCPs in football felt players sometimes (or more frequently) under-reported their symptoms in order to remain on the field. This could either be through poor knowledge of concussion, or poor attitude towards concussion. The responsibility of educating players and increasing their knowledge is not clear. Gouttebarge *et al*^20^ found that while the HCPs surveyed were generally in favour of annual concussion education for players, who should provide that education was split between club and national governing body. However, a systematic review by Baker *et al*^21^ highlights that increasing player knowledge does not necessarily lead to safer attitudes towards concussion or on-pitch behaviours, especially if it is considered an important game. Players suffering from a head injury may not be able to accurately self-assess, or make safe decisions, due to the injury regardless of education or attitude. An over-reliance on players reporting is likely to lead to players remaining on the field with a concussion, presenting a major challenge to HCPs in the absence of an objective measure of the injury.

A form of gamesmanship in football labelled ‘diving’ or ‘feigning injury’ was highlighted by the HCPs in this study. This poses a challenge to HCPs to quickly determine if the player is truly injured as they may exaggerate signs of concussion in an attempt to win a foul. Knowing that the referee will stop the game immediately for a head injury, there is the possibility for tactically feigning injury and interrupting the flow of the game. Comparisons were drawn to the rules in Rugby union where HCPs can immediately enter the field and assess an injured player without stopping the game, so there is no incentive to feign injury. The interruptions to the game in football to assess any suspected injury may help to explain why HCPs in this study can feel under pressure from the crowd or referee to make a quick decision as to whether a player is truly injured. In an attempt to prevent players feigning injury, the International Football Association Board introduced a rule to the 2024/2025 season, where a player must leave the field if they are treated by a HCP and can only return once allowed by the referee.^22^ During this time, their team is at a numerical disadvantage, limiting the tactical advantage of feigning an injury.

Observing how a player reacts following a suspected concussive injury, and seeing the mechanism of injury was very important to the decision-making process of on-pitch HCPs. The exaggeration of symptoms may give a false initial opinion of the injury severity which would result in the need to check for other injuries. Some of the HCPs interviewed emphasised the importance of having instantaneous video replay of the mechanism of injury and being able to observe the players’ reaction. Video replay was pivotal in assisting team doctors in the 2015 Rugby World Cup where it identified 24 head contacts and aided the decision making of 10 of the 39 concussions throughout the tournament.^23^ However, video replay is not available at all stadia in professional football. Increasing the availability of video replay or introducing concussion spotters at better vantage points than the technical area would improve football head injury assessments, especially at lower levels of the professional game. This recommendation echoes that of Gouttebarge *et al*^7^ and Aghababaeian and Yazdi^24^ who both highlighted the importance of video replays.

Football HCPs interviewed in this study highlighted the importance of their relationships with the players and using their clinical judgement to recognise when a potentially injured player is not acting normally. The on-pitch procedure for identifying concussion was described as highly subjective, often with HCPs using their gut instinct to decide if a potentially concussed player was ‘acting normally’. Currently, on-pitch assessments are limited in their objectivity with more appropriate resources and space required to conduct off-pitch assessments in the SCAT6. Subtests of the SCAT-6, such as Maddocks questions and pupillary light reflex responses, were reported to be commonly used as they test fundamental neurocognitive and oculomotor responses but also give indications of the players’ demeanour and ability to follow instructions. Being familiar with players’ normal behaviour is key for HCPs, which can present opportunities where this is possible, but major challenges in instances where it is not.

This study is underpinned by a critical realist framework, which was used to guide our findings. Critical realism focuses on finding the causal mechanisms and environmental variables that lead to observable experiences.^14^ On-pitch decision making for football HCPs is influenced by professional norms, institutional frameworks and interpersonal relationships. The adoption of this perspective facilitated the theorisation of the mechanisms that enable and constrain effective concussion management, moving beyond an account of participants’ experiences.

An area for future research could be developing a quantifiable multimodal assessment for the on–field detection of neurocognitive changes associated with concussion pitch-side. Given the challenges imposed by the current rules of football, that the player may not be temporarily substituted, efforts should be made to improve the information available to the HCPs who are required to decide if a player needs to be permanently removed from play following a head collision event.

### Limitations

While we believe saturation was established, all participants were recruited from the English football leagues. With football being a worldwide sport, future research could emulate this interview on a global scale to deepen the understanding of on-pitch assessment procedures and factors that may inform clinical decision-making for removal from play following a suspected head injury worldwide.

The authors acknowledge that concepts such as saturation and trustworthiness are points of contention within reflexive thematic analysis; therefore, it should be noted that these terms are used to convey sufficient data depth and analytic transparency rather than fixed endpoints of coding.^25 26^ To maintain analytic quality, the framework outlined in the work of Braun and Clarke^26^ was employed to cross-reference methodological and narrative considerations.

## Conclusions

The challenges faced by HCPs in football when deciding whether or not to remove a player following a head impact are: not having enough time to complete a thorough assessment or see symptoms evolve; not being able to see the mechanism of injury and being reliant on subjective on-pitch assessments. Based on the current results, research should focus on developing multimodal testing batteries to detect concussion on-pitch, while football should consider implementing video analysis or concussion spotters at all levels of the game and introducing temporary concussion substitutes to facilitate concussion assessments in line with the recommendations outlined by the consensus statement on concussion in sport guidelines.^1^

### Clinical implications

On-pitch identification of concussion injury in professional football can be improved by:

- Providing HCPs with a video replay of the incident.
- Increasing the 3 min assessment window and moving this to an off-pitch location.
- Promoting familiarity of HCPs with players, which improves subjective assessment of symptoms.

## Data Availability

No data are available.
